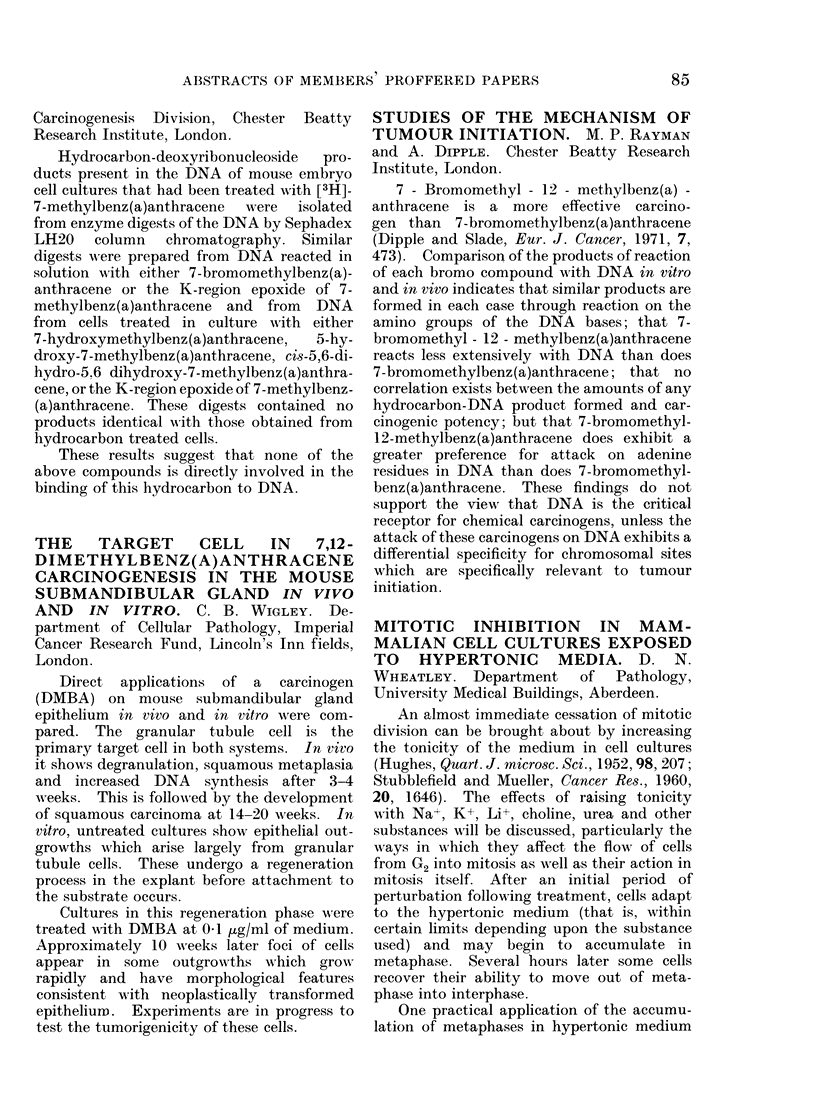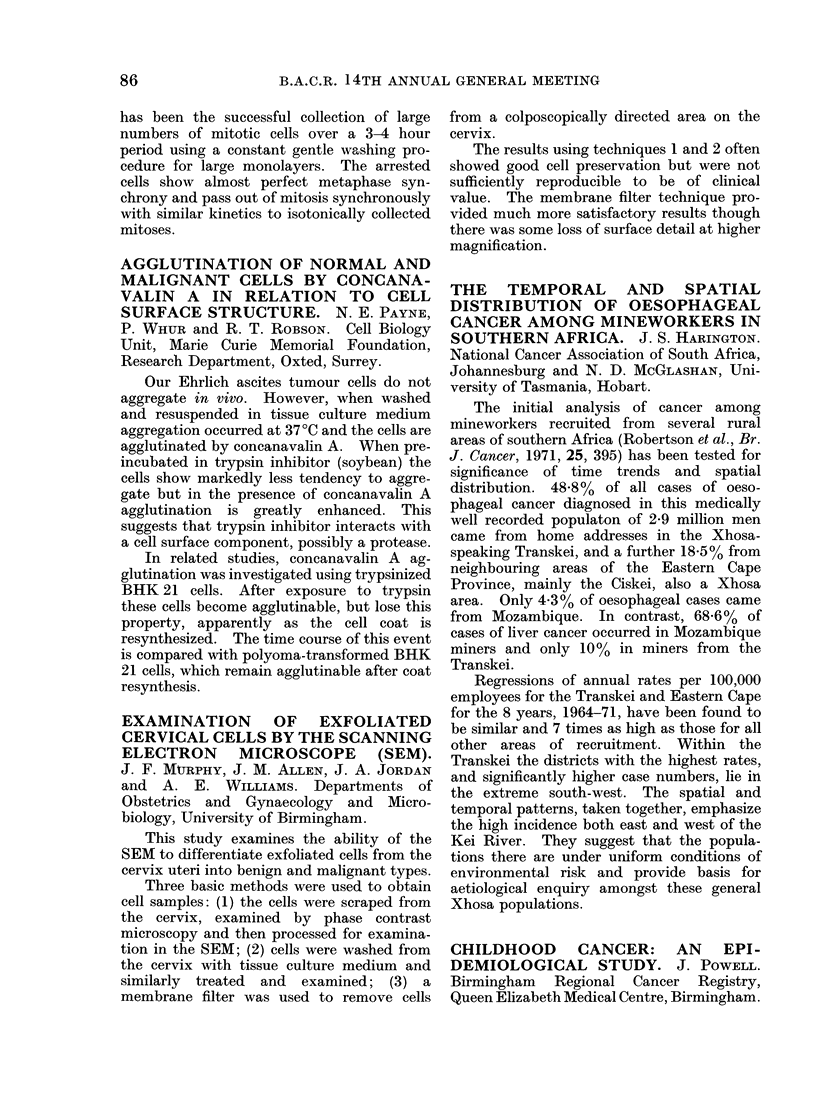# Mitotic inhibition in mammalian cell cultures exposed to hypertonic media.

**DOI:** 10.1038/bjc.1973.106

**Published:** 1973-07

**Authors:** D. N. Wheatley


**MITOTIC INHIBITION IN MAMMALIAN CELL CULTURES EXPOSED TO HYPERTONIC MEDIA.** D. N. WHEATLEY. Department of Pathology, University Medical Buildings, Aberdeen.

An almost immediate cessation of mitotic division can be brought about by increasing the tonicity of the medium in cell cultures (Hughes, *Quart. J. microsc. Sci.*, 1952, **98**, 207; Stubblefield and Mueller, *Cancer Res.*, 1960, **20**, 1646). The effects of raising tonicity with $Na^+$, $K^+$, $Li^+$, choline, urea and other substances will be discussed, particularly the ways in which they affect the flow of cells from $G_2$ into mitosis as well as their action in mitosis itself. After an initial period of perturbation following treatment, cells adapt to the hypertonic medium (that is, within certain limits depending upon the substance used) and may begin to accumulate in metaphase. Several hours later some cells recover their ability to move out of metaphase into interphase.

One practical application of the accumulation of metaphases in hypertonic medium has been the successful collection of large numbers of mitotic cells over a 3–4 hour period using a constant gentle washing procedure for large monolayers. The arrested cells show almost perfect metaphase synchrony and pass out of mitosis synchronously with similar kinetics to isotonically collected mitoses.